# Pregnancy Complicated with Severe Recurrent Aortic Coarctation: A Case Report

**DOI:** 10.1155/2012/865035

**Published:** 2012-10-24

**Authors:** Celal Yavuz, Hatice Ender Soydinc, Güven Tekbaş, Oguz Karahan

**Affiliations:** ^1^Department of Cardiovascular Surgery, Faculty of Medicine, Dicle University, 21280 Diyarbakir, Turkey; ^2^Department of Obstetrics and Gynaecology, Faculty of Medicine, Dicle University, 21280 Diyarbakir, Turkey; ^3^Department of Radiology, Faculty of Medicine, Dicle University, 21280 Diyarbakir, Turkey

## Abstract

A 23-year-old primigravida was referred to our clinic for evaluation of high blood pressure (BP) in her 16th week of gestation. She had an operation to repair congenital aortic coarctation and patent ductus arteriosus 8 years ago. On physical examination the blood pressure in upper extremity was 155/95 and in lower extremity was 90/55 mmHg, and heart rate was 93 beats/min. Transthoracic echocardiography showed narrowing of the descending aorta, the diameter of the aortic arch was 10.60 mm and an echocardiographic gradient was 96 mmHg. During the pregnancy (from 16 weeks to 38 weeks) BP was regulated with metoprolol. Cesarean section delivery was applied at 38 weeks of gestation. There was no complication in postpartum period. Spinal anesthesia application was used for caesarean section intervention and healthy female baby was delivered with the APGAR scores of 10/10. Herein the diagnosis of aortic coarctation is reviewed and the management when found during pregnancy is discussed.

## 1. Introduction

Aortic coarctation (AoCo) accounts for 5–8% of all congenital heart defects. In recent years alternative treatments of AoCo have been developed. Therefore, the number of women who reach childbearing age with AoCo has been increased and this group now represents the majority of women with congenital heart disease during pregnancy [1]. Nonetheless, the disorder is important to recognize, because dangerous complications of the pregnancy thereby can be minimized. Herein, we report the case of a symptomatic pregnant in whom significant AoCo was successfully managed. Cesarean section delivery was applied at 38 weeks of gestation.

## 2. Case Report

 A 23-year-old primigravida was referred to our clinic with the complaint of headache, fatigue, and high blood pressure in 16 weeks of gestation. Patient's functional capacity was in NYHA II. In medical history, she had an operation to repair congenital Aortic coarctation with a pericardial patch and also she had a patent ductus arteriosus operation at the age of 15 years old (patent ductus arteriosus was identified and carefully dissected after left anterolateral thoracotomy. The ductus was dissected out and ligated with nonabsorbable sutures. Coarctation segment (distal subclavian segment of descending aorta-poststenotic dilatation) was repaired by pericardial patch plasty). Moreover, she had undergone a repair operation of aortic aneurysm at the age of 21 years old (the aneurysm formation onto previous patch plasty segment of thoracic aorta (with 5.4 cm diameter) was detected in the patient. The aneurysm was resected and replaced with a simple interposition of Dacron graft). When she came to the clinic she did not use any medication. Physical examination revealed a 65 mmHg difference in blood pressure between the upper and lower extremities (upper extremity was 155/95 and lower extremity was 90/55 mmHg, resp.) and heart rate was 93 beats/min. 1-2/6 degree systolic murmur was detected in the examination of cardiovascular system. The femoral and popliteal pulses were palpated bilaterally but they were weak. Biochemical laboratory tests were normal. Her electrocardiogram was normal. 

**Figure 1:**
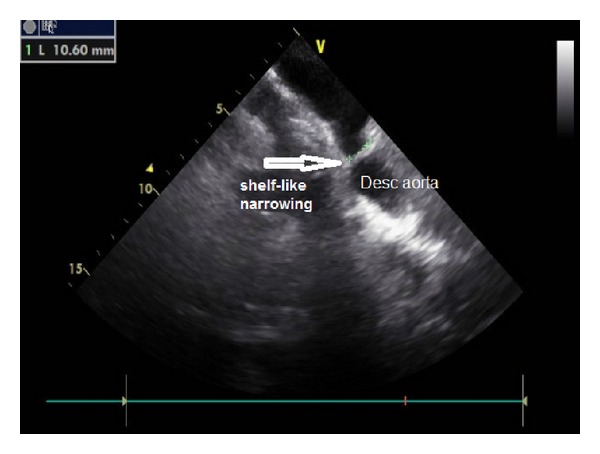
Transthoracic echocardiography showed narrowing of the descending aorta; diameter of the aortic arch was 10.60 mm.

**Figure 2:**
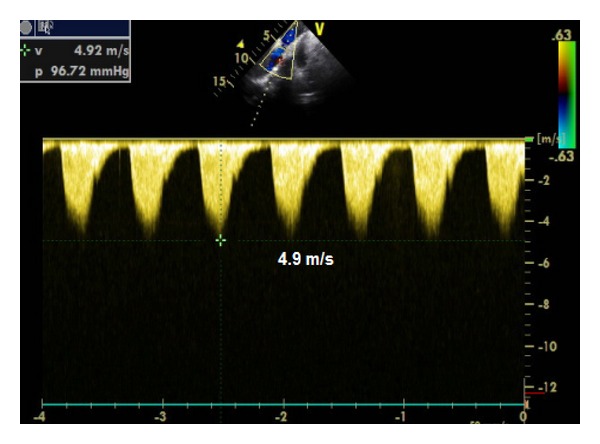
A Doppler echocardiographic gradient was 96 mmHg.

**Figure 3:**
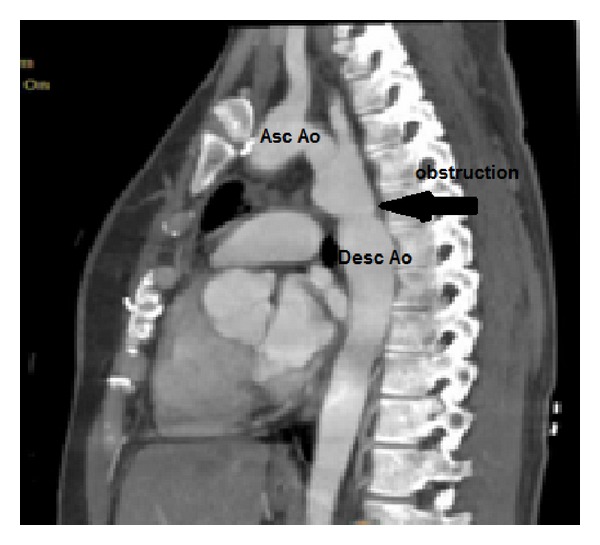
Computed tomography angiography demonstrates the postductal coarctation of the aorta. The descending aortic arch is tortuous and the diminutive left subclavian artery arises proximal to the coarctation.

Transthoracic echocardiography showed normal echocardiographic findings, long axis from the suprasternal notch demonstrated shelf-like narrowing of the descending aorta, diameter of the aortic arch was 10.60 mm (Figure 1), and continuous wave Doppler of the descending aorta revealed a peak velocity of 4.92 m/s and maximal systolic pressure gradient was 96 mmHg (Figure 2). Transthoracic echocardiography showed that there were no other congenital heart disease especially bicuspid aortic valve and mitral valve malformations. During the pregnancy (from 16 weeks to 38 weeks) BP was regulated with metoprolol 50 mg and also Doppler gradient 96 mmHg was stable on serial echocardiograms. Cesarean section delivery was applied at 38 weeks of gestation. No aortic rupture or dissection was observed during pregnancy and labor. There was no complication in postpartum period. The child was born healthy. The children were examined by pediatric cardiologist and she had no a congenital heart defect. Computed tomography (CT) was performed to evaluate coarctation anatomy at the postpartum period. CT angiography revealed elongation and narrowing of the descending aorta. The transverse diameter of the proximal aortic arch was narrowed from 22 mm to 11 mm (Figure 3). Blood pressure is under control at postpartum period with metoprolol 50 mg. Endovascular aortic stent was applied into Dacron repaired segment of aorta after the 6th month of labor. 

## 3. Discussion

Typically AoCo is located in the area where the ductus arteriosus inserted and only in rare cases occurs ectopically (ascending, descending, or abdominal aorta) [1]. Classically, AoCo is poorly tolerated during pregnancy due to associated with a risk of acute aortopathy. In contrast to common misconception the majority of women with AoCo do well during pregnancy [2]. However, AoCo may cause some problems during pregnancy even after surgical or stent repair because of hypertensive disorders [3]. Vriend et al. have reported on 126 pregnancies in 54 women after repair of AoCo, which renders it the largest reported series thus far [3]. The present study showed that pregnancy is well tolerated in postcoarctectomy patients [3].

However, an excess of miscarriages and frequent occurrence of hypertensive disorders of pregnancy were observed [4]. These patients should be evaluated for early detection and prevention of obstetrical and/or cardiovascular complications such as sustained hypertension, aortic root dilatation, or recoarctation. Saidi and coworkers reported that the risk of aneurysm formation and the development of systemic hypertension are uncertain in patients with aortic coarctation. They added that in women with an arm-to-leg blood pressure gradient of <20 mm Hg after AoCo repair, pregnancy is successful [5].

Hypertension worsens in some patients and the spontaneous abortion rate is increased [2]. From the third to the seventh month of pregnancy circulation is successively accelerated, and blood volume and cardiac output increase by 30 to 50%. The blood pressure balance shows sudden changes in the seventh month of pregnancy, despite the average blood pressure regular. Thus, this period is the first critical period for the AoCo patient. The second critical period is labour itself, because blood pressure and cardiac work increase by about 20% at the peak of each uterine contraction. Vriend and et al. reported on 126 pregnancies in 54 women after repair of aortic coarctation; the study shows that pregnancy is well tolerated in postcoarctectomy [3]. Women with significant AoCo contemplating pregnancy should undergo repair before pregnancy. For all these patients obstetricians, anaesthetists, and cardiologists should closely cooperate, ideally on the same site. Women with a history of coarctation repair, who contemplate pregnancy, should have haemodynamic assessment and genetic counseling, preferably before conception. Endocarditis prophylaxis was not administered because current guidelines do not recommend this in patients with AoCo.

## 4. Conclusion

 We reported a pregnant woman complicated with severe AoCo who resulted in successful delivery without cardiovascular complication.
